# Associations between skeletal muscle mass and elevated blood pressure are independent of body fat: a cross-sectional study in young adult women of African ancestry

**DOI:** 10.1017/S0007114525000029

**Published:** 2025-02-14

**Authors:** Siphiwe N. Dlamini, Shane A. Norris, Lisa K. Micklesfield

**Affiliations:** 1 School of Physiology, Faculty of Health Sciences, University of the Witwatersrand, Johannesburg, South Africa; 2 SAMRC/Wits Developmental Pathways for Health Research Unit, Faculty of Health Sciences, University of the Witwatersrand, Johannesburg, South Africa; 3 School of Human Development and Health, University of Southampton, Southampton, UK

**Keywords:** Skeletal muscle mass, Body fat mass, Systolic blood pressure, Diastolic blood pressure

## Abstract

Although research on the relationship between lean body mass and blood pressure (BP) has been inconsistent, most studies reported that measures of lean body mass are associated with a higher risk of hypertension. We explored relationships between body composition (fat and skeletal muscle mass) and BP in 1162 young adult African women. Dual-energy X-ray absorptiometry-derived measures of whole-body, central and arm fat mass were associated with higher systolic and diastolic BP, while leg fat percentage was associated with lower systolic and diastolic BP. However, only the associations with diastolic BP remained after adjusting for appendicular skeletal muscle mass (ASM). ASM was associated with higher systolic and diastolic BP, before and after adjusting for whole-body fat percentage and visceral adipose tissue. While there was no overlap in targeted proteomics of BP and body composition, REN was lower in the elevated BP than the normal BP group and was inversely associated with diastolic BP (false rate discovery adjusted *P*< 0·050). Several proteins were positively associated with both visceral adipose tissue and ASM (LEP, FABP4, IL6 and GGH) and negatively associated with both visceral adipose tissue and ASM (ACAN, CELA3A, PLA2G1B and NCAM1). NOTCH3, ART3, COL1A1, DKK3, ENG, NPTXR, AMY2B and CNTN1 were associated with lower visceral adipose tissue only, and IGFBP1 was associated with lower ASM only. While the associations between body fat and BP were not independent of skeletal muscle mass, the associations between muscle mass and BP were independent of overall and central adiposity in young adult African women. Future interventions targeting muscle mass should also monitor BP in this population.

The major risk factors for CVD include smoking, dyslipidaemia and diabetes, but hypertension – protracted elevated blood pressure (BP) – is the most prevalent and strongest CVD predictor^(1)^. In the past few decades, there has been a significant increase in the global prevalence of hypertension, with low- and middle-income countries being disproportionally affected^(2)^. This increase is largely due to risk factors such as unhealthy diets, insufficient physical activity and excess weight gain due to poorly planned urbanisation^(3)^. Among these risk factors, excess weight gain (as seen in obesity) accounts for at least 65 % of all primary hypertension cases^(4)^.

Body composition mainly comprises adipose tissue (body fat), fat-free tissue (lean body mass) and extracellular water^(5)^, but excess weight gain is generally characterised by accumulation of more fat than lean mass^(6)^. Skeletal muscles comprise a large portion of lean body mass and are well-acknowledged determinants of overall health. This is partly because skeletal muscles play key roles in regulating systemic metabolism, energy expenditure and homeostasis^(7–9)^ and also serve as the primary site of glucose uptake^(10)^. Accordingly, individuals with a combination of high body fat mass and low skeletal muscle mass are at the highest risk of developing CVD^(11)^. Such observations have led to the hypothesis that while body fat mass associates with increased hypertension risk^(12–14)^, lean body mass is protective against hypertension^(15)^. The associations between body fat mass and hypertension risk are likely due to unfavourable body fat distribution, including high visceral adipose tissue (VAT) and low subcutaneous adipose tissue (SAT). This is because SAT has less deleterious effects than VAT, mainly due to differences in receptor expression, adipocyte size, secretome and other metabolic features^(16)^. The deleterious effects of VAT on hypertension risk are also attributed to its location in the intra-abdominal cavities and around internal organs, where it releases active proteins that influence key metabolic functions in organs like the pancreas, liver and heart^(17–19)^.

Conversely, studies that have investigated associations between measures of lean body mass and BP have been inconsistent. Some findings have shown that lean body mass was lower in individuals at higher risk of hypertension, supporting the hypothesis that lean body mass is protective against hypertension^(9,20)^. However, most studies have reported the opposite direction of association, regardless of whether an index or absolute measure of lean mass was used^(13,18,21,22)^. The conflicting associations between lean body mass and BP may be attributed to differences in body fat distribution, age, metabolic health, sex and ethnic variations. Positive associations are often reported in populations with higher VAT and older adults with cardiometabolic diseases, while negative associations were more common in younger, healthier populations with higher SAT^(9,13,17)^. Additionally, methodological differences in measurement techniques and study design also contributed to these varying findings. For example, the formula used by Han *et al.* to estimate muscle mass was calculated from anthropometric measurements. The study utilised measurements such as body weight, height and other relevant anthropometric data to derive the muscle mass estimation formula^(9)^. We have demonstrated that in men and women of African ancestry, living in urban Soweto, South Africa, the ratio of adiposity (dual-energy X-ray absorptiometry (DXA)-derived fat mass) to lean mass (DXA-derived fat-free soft tissue mass) was the most strongly associated with hypertension^(23)^. Based on the observation that most populations with high adiposity report a positive association between muscle mass and BP, we hypothesised that young adult women of African ancestry would also show a positive association due to their generally high body fat mass^(23)^. Studies in European children and adolescents have shown that lean body mass was associated with BP independent of body fat and that lean body mass was a stronger predictor of BP compared with body fat mass^(24,25)^. These relationships were recently confirmed in a multi-ethnic study of young and middle-aged adults, which demonstrated that both skeletal muscle mass and body fat independently associate with higher systolic BP^(22)^.

Studying associations between body composition and BP in populations of African ancestry is crucial due to the notable ethnic disparities in both body composition and cardiometabolic disease risk^(26)^. For example, women of African ancestry typically have higher body fat mass and lower VAT and are at a higher risk of developing diseases such as hypertension and type 2 diabetes than their European counterparts^(27,28)^. Further, investigating protein biomarkers of body composition, through targeted proteomics, has the potential to yield important insights into the biological mechanisms involved in the association between body composition and hypertension risk^(29–31)^. This is because the human proteome serves as an intermediate measurement between the genetics and the environment and the complex disease risk^(32)^. Therefore, identifying protein biomarkers that overlap between measures of skeletal muscle mass, body fat mass and BP could elucidate the pathways through which body composition influences BP regulation and the risk of hypertension.

Therefore, the primary aim of this study was to investigate whether body fat mass and its distribution and skeletal muscle mass are associated with BP in young adult women of African ancestry and whether these associations are independent of the other. We also aimed to use a targeted proteomics approach to gain some insights into the potential biological mechanisms that may explain these relationships.

## Experimental methods

### Study population

The present study included participants from the BUKHALI (BUilding Knowledge and a foundation for HeALthy lIfe trajectories) trial, the South African component of the Healthy Life Trajectories Initiative (HeLTI) international collaboration, which was described elsewhere^(33)^. Briefly, the BUKHALI study is a randomised trial testing the efficacy of micronutrient supplements and behaviour change interventions to improve diet and physical activity during preconception and health during pregnancy, reduce perinatal depression and increase exclusive breastfeeding and improve parental nurturing care. Inclusion criteria in BUKHALI were women aged 18–28 years at baseline and residing in Soweto – an urban township in Johannesburg South Africa. Exclusion criteria were women with type-I diabetes mellitus, cancer or epilepsy, intellectual disability or those who were not able or willing to provide consent. Data and samples used in the present cross-sectional study were collected between June 2018 and June 2019 and were the baseline data from the pilot phase of the BUKHALI trial, comprising 1168 participants with body composition data^(34)^. Data included socio-demographic, lifestyle and health questionnaire data, anthropometry, peripheral BP and DXA-derived body composition.

### Questionnaire data

Socio-demographic, health and lifestyle data were collected by a Computer-Assisted Personal Interview mode. Age was confirmed using dates of birth from respective national identity documents. Participants were asked if they currently smoked and were subsequently classified into current smokers and non-smokers. Likewise, the participants were classified as alcohol consumers or non-alcohol consumers. Participants were asked to bring all their medications to the research centre for recording chronic medication use. To determine HIV status, each participant was asked if they had ever tested HIV positive, and those who replied ‘Yes’ were classified as living with HIV.

### Anthropometry, body composition and blood pressure

All anthropometric values were measured in triplicate, and then the mean values were used in the analyses. The participants were wearing light clothing and no shoes when height and weight were measured. A wall-mounted stadiometer (Holtain) was used to measure height to the nearest 0·1 cm, and a calibrated digital scale (SECA) was used to measure weight to the nearest 0·1 kg. BMI was then calculated as weight (kg) divided by height squared (m^2^). Waist circumference was measured halfway between the iliac crest in the midaxillary plane and the lowest rib margin, using a soft measuring tape and to the nearest 0·1 cm.

A QDR 4500A DXA machine (Hologic) was used to measure whole-body composition, including body fat mass, lean mass and bone mineral content. The DXA data were then analysed with APEX software version 13.4.2.3 (Hologic). Subsequently, fat-free soft tissue mass (FFSTM) was calculated as lean mass minus bone mineral content and used as a proxy for skeletal muscle mass. The FM/FFSTM ratio was calculated by dividing whole-body fat mass by FFSTM. Total appendicular skeletal muscle mass (ASM) was calculated as a sum of skeletal muscle mass of both arms and legs, and the ASM index is calculated as ASM divided by height squared (kg/m^2^). DXA-derived fat variables included sub-total (total body minus head) fat mass and as a percentage of the whole body, leg and arm fat (kg and % of sub-total body fat) and VAT and SAT.

Systolic and diastolic BP were measured on the left arm using an automated BP machine (Omicron M6) and appropriately sized cuffs. Participants were required to be seated for at least 5 min after which three BP readings were taken at 2-min intervals. The average of the second and third readings was used in the analyses.

### Power calculation and sample selection

Our statistical power estimate was based on the associations between ASM (the main predictor) and BP (the main outcome). The effect size estimate was from a recently reported association between ASM and systolic BP in adult men of mixed ancestry, which suggested an effect size of 0·13^(22)^. Using the ‘pwr.f2.test’ function from the ‘pwr’ package in R, we found that a total of sixty-three participants would be required to reach 80 % power in a simple linear regression statistical model, given an *α* level of 0·05, in a two-sided test. Power analysis is typically not conducted for OLINK (the proteomics method used in this study) for several reasons including the exploratory nature and the use of normalised protein expression values – which are logarithmic and based on internal controls and inter-sample comparison^(35)^. For these types of proteomic studies, power is maximised by having equal sample sizes in both groups, and this is known as a balanced design^(35)^. Accordingly, as the prevalence of hypertension was relatively low among young adults in the BUKHALI cohort, we maximised our statistical power by ensuring that 50 % of the samples had elevated BP or hypertension.

**Fig. 1.**
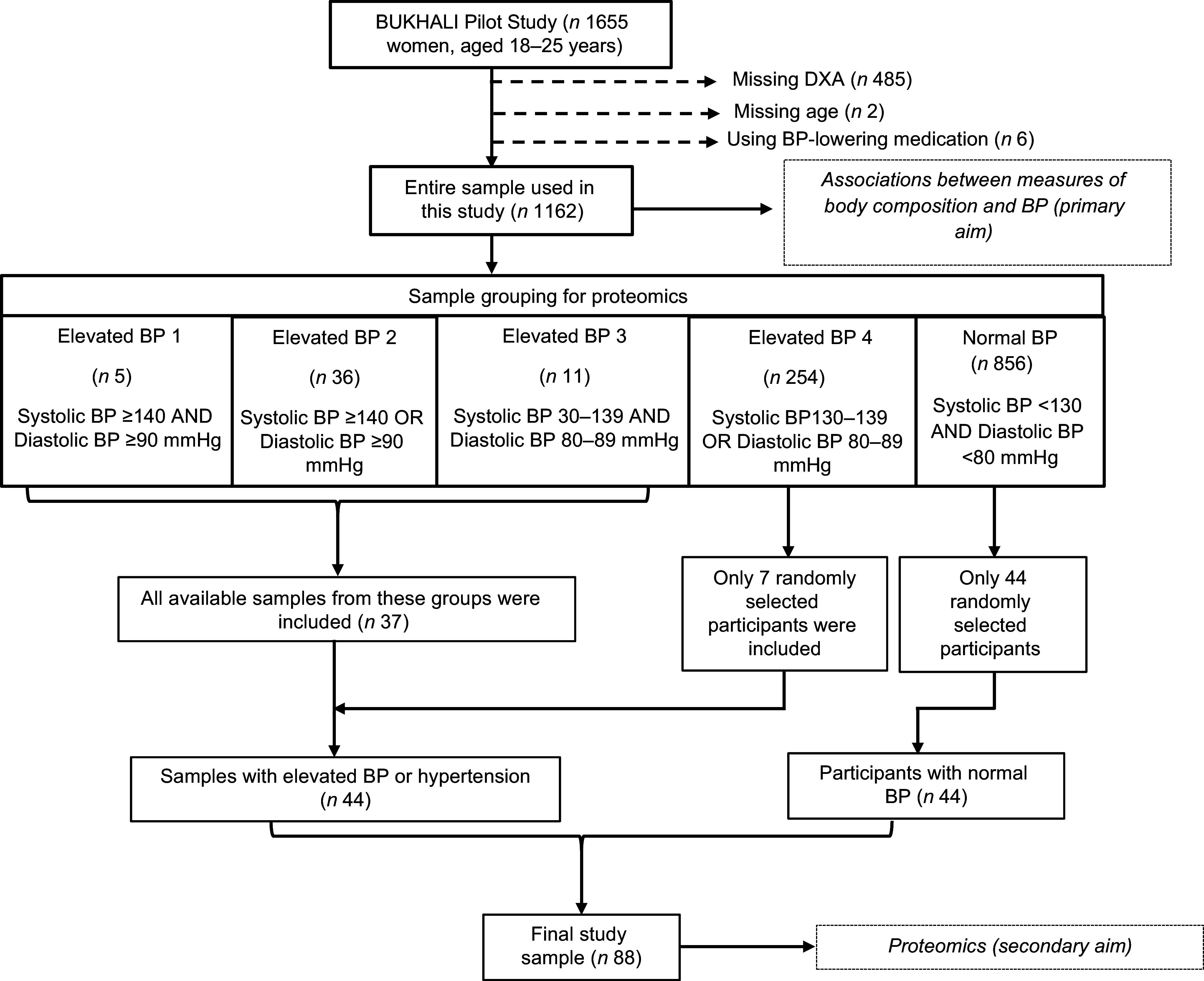
Selection of the participants for proteomic analysis from the BUKHALI (BUilding Knowledge and a foundation for HeALthy lIfe trajectories) cohort. DXA, dual-energy X-ray absorptiometry; BP, blood pressure.

Figure 1 summarises the steps used to select the samples and participants included in the present study. To test the associations between measures of body composition and BP, all participants from the BUKHALI pilot (baseline) were considered (*n* 1655). From that sampling frame, we removed 485 participants because of missing DXA measurements and two participants because of missing age values. Six participants were using BP-lowering medication and were excluded. Therefore, a total of 1162 participants were included when testing the associations between measures of body composition and BP (the primary aim of the study).

The sample plate for proteomics could accommodate up to eighty-eight samples. Hence, for proteomics (secondary aim), we selected a total of eighty-eight samples, of which forty-four (50 %) had elevated BP or hypertension, using the steps also summarised in Fig. 1. First, the entire sample (*n* 1162) was divided into five groups based on their BP measurements as follows. ‘Elevated BP 1’ were participants with systolic BP ≥ 140 and diastolic BP ≥ 90 mmHg (*n* 5). ‘Elevated BP 2’ were participants with systolic BP ≥ 140 or diastolic BP ≥ 90 mmHg (*n* 36). ‘Elevated BP 3’ included participants with systolic BP in the range of 130–139 mmHg and diastolic BP in the range of 80–89 mmHg (*n* 11). ‘Elevated BP 4’ included participants with systolic BP in the range of 130–139 mmHg or diastolic BP in the range of 80–89 mmHg (*n* 254). ‘Normal BP’ included participants with systolic BP in the range of < 130 mmHg and diastolic BP in the range of < 80 mmHg (*n* 856).

Notably, two and thirteen samples from the elevated BP groups 1 and 2, respectively, were excluded because of insufficient plasma volumes. To select eighty-eight samples, we first included all available samples from elevated BP groups 1 to 3 (*n* 3 + 23 + 11 = 37). Thereafter, we used a sampling computer programme (‘sample’ function in R), to randomly select seven participants from the elevated BP 4 group and forty-four participants from the normal BP group. Ultimately, the samples selected from the four elevated BP groups were combined to form one group called ‘elevated BP’ (*n* 44).

### Blood sampling and proteomic analysis

Standard venepuncture techniques were used to collect random (non-fasting) blood samples, which were used for the determination of plasma protein biomarkers. Targeted proteomics analyses were conducted at the BioXpedia laboratory services using OLINK proteomics panels. OLINK proteomic analyses use proximity extension assay technology, which is a 96-plex immunoassay for high throughput detection of protein biomarkers in plasma samples. The principles behind this method are described elsewhere (www.bioxpedia.com/olink-proteomics). For the present study, we selected the Explore Cardiometabolic Panel I service that included 366 cardiometabolic biomarkers. The proteomic data were reported as normalised protein expression values. An normalised protein expression value is OLINK’s relative protein quantification unit on the log2 scale. In the present study, three protein biomarkers (BMP6, EPHX2 and PGLYRP1) were excluded because they failed OLINK’s batch release quality control criteria. Therefore, only 363 cardiometabolic biomarkers were included in the statistical analysis.

### Statistical analysis

Statistical analyses were conducted in R version 4.2.3^(36)^. The normality of the continuous variables was assessed using a Shapiro–Wilk test. Continuous variables were not normally distributed and were thus presented as median (25th–75th percentiles) when comparing basic characteristics. Accordingly, a Wilcoxon rank-sum test was used to assess statistical differences between the normal and elevated BP groups. Categorical variables were presented as observations and percentages: *n* (%), and a *χ* ^2^ test was used to assess statistical differences between normal and elevated BP groups. Associations between continuous predictors and outcomes were tested using linear regressions. For associations between measures of body fat distribution (VAT, SAT and sub-total fat mass %, arm and leg fat %) and systolic and diastolic BP, three sets of regression models were tested. The first set of regression models was not adjusted for any potential confounders, while the second set was adjusted for the main confounders (age, height, smoking, alcohol and known HIV status) only. In the third set of models, ASM was included as an additional confounder. For the associations of the FM/FFSTM ratio, only the first (unadjusted) and second models (adjusted for the main founders) were conducted. Conversely, in the associations of ASM and ASM index with systolic and diastolic BP, four sets of regression models were tested: (1) unadjusted, (2) adjusted for the main confounders (age, height, smoking, alcohol, known HIV status) only, (3) adjusted for the main confounders and sub-total body fat percentage and (4) adjusted for the main confounders and VAT. Importantly, height was excluded as a confounder in the models where the ASM index was the predictor because this introduced multicollinearity. Multicollinearity was ruled out in each adjusted model by evaluating the variance inflation factor values, all of which were below 2·0. Prior to inclusion in the models, systolic and diastolic BP values were mathematically transformed using a Standardised asinh(x) in R, to make the data normally distributed.

The ‘olink_ttest’ function of the Olink® Analyze R package was used to test differences in the protein biomarkers between the normal and elevated BP groups of the subsample (*n* 44 participants per group). Additionally, we used linear regression models to test associations between all protein biomarkers with each of the following continuous outcomes: systolic BP, diastolic BP, VAT and ASM. Prior to inclusion as an outcome in the linear regression models, VAT and ASM were mathematically transformed using a box-cox function in R, to make the data normally distributed. Each regression model was first run without any confounder (unadjusted), and then in the second set, the models were adjusted for the main confounders: age, height, smoking, alcohol and HIV status. The Benjamini–Hochberg false discovery rate (FDR) was used to control for multiple testing, and the FDR-adjusted *P* value < 0·050 was considered sufficient evidence of association in the proteomic analysis. The FDR-adjusted *P* values were only reported for the adjusted models (adjusted for the main confounders).

## Results

### Basic characteristics of the study sample

Table 1 shows the basic characteristics of the study sample and compares participants with elevated BP to their normal BP counterparts. Participants with elevated BP were older and had higher weight, BMI, waist circumference, VAT and SAT, all measures of skeletal muscle mass (arms, legs and total, ASM index), and all measures of fat mass (sub-total, arm and leg fat mass and FM/FFSTM), compared with those who had normal BP (all *P*< 0·01). Compared with the normal BP group, whole-body fat percentage was higher in the elevated BP group (*P*< 0·001). Regarding measures of fat distribution, which were in relation to whole-body fat, arm fat percentage was higher (both *P*< 0·02), while leg fat percentage was lower (both *P*< 0·001) in participants with elevated BP, compared with their normal BP peers.

**Table 1.** Basic characteristics of the study sample (Median values and 25th–75th percentiles; numbers and percentages)

	All (*n* 1162)		Normal BP (*n* 856)		Elevated BP (*n* 306)		
	Median	25th–75th percentiles	Median	25th–75th percentiles	Median	25th–75th percentiles	*P*
Blood pressure							
Systolic blood pressure (mmHg)	107·0	99·8–114·3	104·0	97·7–110·3	115·0	110·3–122·3	2·20 × 10^−16^
Diastolic blood pressure (mmHg)	75·0	70·0–80·0	72·7	68·3–76·3	83·3	81·3–86·3	2·20 × 10^−16^
Age (years)	21	19–23	21	19–23	21	20–23	0·005
Anthropometry							
Height (cm)	159·5	155·5–163·5	159·4	155·4–163·5	159·8	156·2–164·1	0·179
Weight (kg)	61·2	53·2–72·1	60·3	52·8–70·5	65·2	55·7–79·8	3·04 × 10^−07^
BMI (kg/m^2^)	24·0	21·0–28·4	23·5	20·7–27·7	25·3	21·6–31·2	1·60 × 10^−06^
Waist circumference (cm)	85·9	76·6–96·9	84·7	75·9–95·0	89·6	78·7–103·2	1·30 × 10^−06^
Dual-energy X-ray absorptiometry							
Body fat							
Sub-total fat mass (kg)	22·9	17·0–30·4	22·0	16·5–29·1	25·4	18·3–35·7	1·41 × 10^−06^
VAT (cm^2^)	58·2	38·2–89·3	54·2	36·9–81·9	67·2	44·9–109·6	6·38 × 10^−08^
SAT (cm^2^)	293·5	200·5–418·7	281·2	194·7–396·2	335·6	228·2–484·6	9·25 × 10^−07^
Leg fat mass (kg)	11·0	8·6–14·1	10·6	8·4–13·5	11·7	9·2–15·5	7·39 × 10^−05^
Arm fat mass (kg)	2·6	1·8–3·6	2·5	1·7–3·4	2·9	1·9–4·2	2·37 × 10^−06^
FM/FFSTM	1·4	1·2–1·8	1·4	1·1–1·7	1·6	1·2–1·9	9·40 × 10^−05^
Body fat % in relation to whole-body fat							
Whole-body fat (%)	37·1	31·8–42·5	36·9	31·3–41·7	39·1	33·3–44·5	4·33 × 10^−05^
Leg fat (%)	48·4	44·8–52·2	48·9	45·3–52·5	46·9	42·6–51·3	4·63 × 10^−07^
Arm fat (%)	11·2	10·1–12·2	11·1	10·0–12·1	11·3	10·3–12·4	0·015
Skeletal muscle mass							
Leg skeletal muscle mass (kg)	12·2	10·8–14·1	12·1	10·7–13·9	12·7	11·2–14·7	1·10 × 10^−04^
Arm skeletal muscle mass (kg)	3·6	3·2–4·0	3·5	3·2–3·9	3·7	3·3–4·3	1·27 × 10^−07^
Total appendicular skeletal muscle mass (kg)	15·8	14·2–18·1	15·7	13·9–17·8	16·4	14·8–18·8	1·54 × 10^−05^
Appendicular skeletal muscle mass index (kg/m^2^)	6·2	5·6–7·0	6·1	5·6–6·9	6·4	5·7 to7·4	5·02 × 10^−05^
	*n*	%	*n*	%	*n*	%	
Lifestyle and medication							
Current smokers (*n* (%))	275·	23·7	212·	24·8	63·	20·6	0·191
Current alcohol drinkers (*n* (%))	791·	68·1	587·	68·6	204·	66·6	0·481
Living with HIV (*n* (%))	58·	5·0	49·	5·7	9·	2·9	0·049

Continuous data presented as median (25th–75th percentiles). Wilcoxon rank-sum test was used to compare the continuous variables, while a *χ* ^2^ test was used to compare the categorical variables. BP, blood pressure; VAT, visceral adipose tissue; SAT, subcutaneous adipose tissue; *n* (%), number of observations (percentage); FM/FFSTM, whole-body fat mass/fat-free soft tissue mass. Android, gynoid, leg and arm percentages were calculated in relation to sub-total fat mass. For example, android fat percentage (%) was calculated as (android fat mass/sub-total fat mass) × 100 %.

Regarding lifestyle factors, there were no differences in the proportion of current smokers and alcohol consumers when comparing participants with elevated BP to their normal counterparts. However, there were fewer participants living with HIV in the elevated BP group compared with the normal BP group (2·9 % *v*. 5·7 %, *P*= 0·049).

### Associations between measures of body composition and blood pressure

**Fig. 2.**
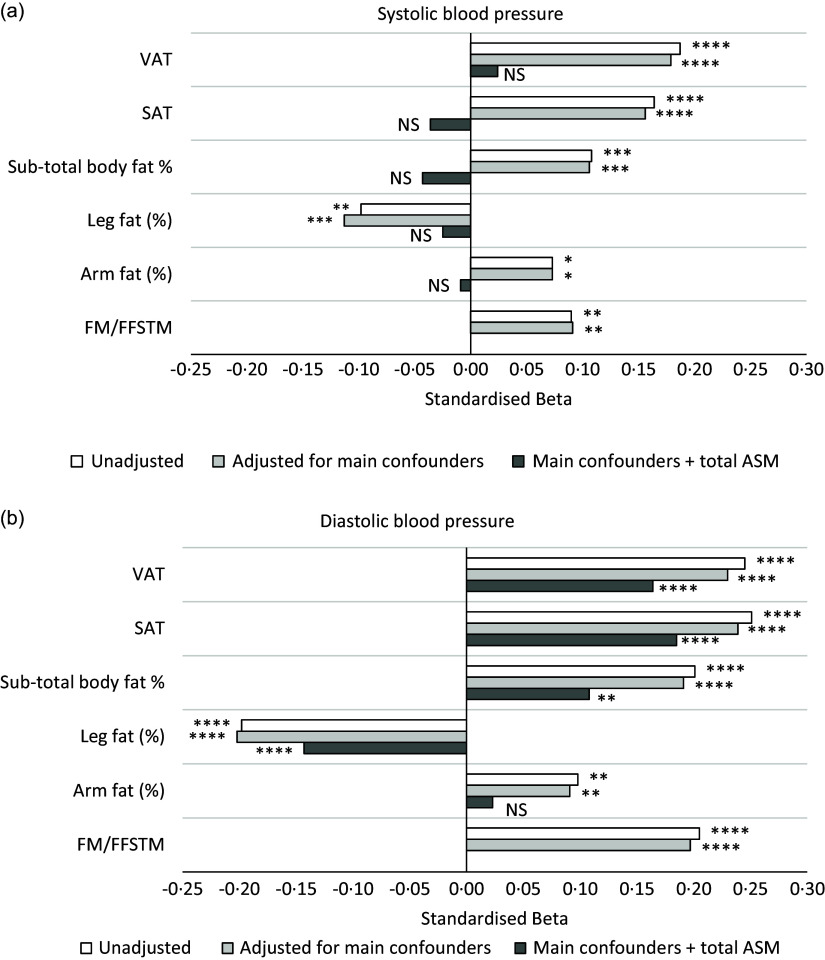
Associations of measures of body fat and body fat distribution (predictors) with systolic (a) and diastolic (b) blood pressure (outcomes). Linear regression models unadjusted; adjusted for main confounders (age, height, smoking, alcohol and HIV status) only; and adjusted for main confounders plus total appendicular skeletal muscle mass (ASM). Systolic and diastolic blood pressure (BP) values were mathematically transformed using a Standardised asinh(x) function in R, prior to inclusion in the models. ****P< 0·0001, ***P< 0·001, **P< 0·01, *P< 0·05, VAT, visceral adipose tissue; SAT, subcutaneous adipose tissue; FM/FFSTM, whole-body fat mass/fat-free soft tissue mass. Leg and arm percentages were calculated in relation to sub-total fat mass. For example, leg fat percentage (%) was calculated as (leg fat mass/sub-total fat mass) × 100.

Figure 2 summarises linear regression models for the associations between measures of body fat distribution and measures of BP (systolic and diastolic BP).

VAT, SAT and sub-total body fat percentage were associated with higher systolic and diastolic BP, even after adjusting for the main confounders (all *P*< 0·001). However, after adjusting for the differences in ASM, the associations only remained for diastolic BP (all *P*< 0·001) and not systolic BP (all *P*> 0·05). Similarly, the FM/FFSTM ratio was associated with higher systolic and diastolic BP, even after adjusting for the main confounders (*P*< 0·001). We also found that arm fat percentage was associated with higher systolic and diastolic BP, even after adjusting for the main confounders (both *P*< 0·05), but not after adjusting for ASM. Converse to the above relationships, leg fat percentage was associated with lower systolic and diastolic BP even after adjusting for the main confounders (all *P*< 0·001). However, only the association between leg fat percentage and lower diastolic BP remained after adjusting for ASM (*P*< 0·001).

**Fig. 3.**
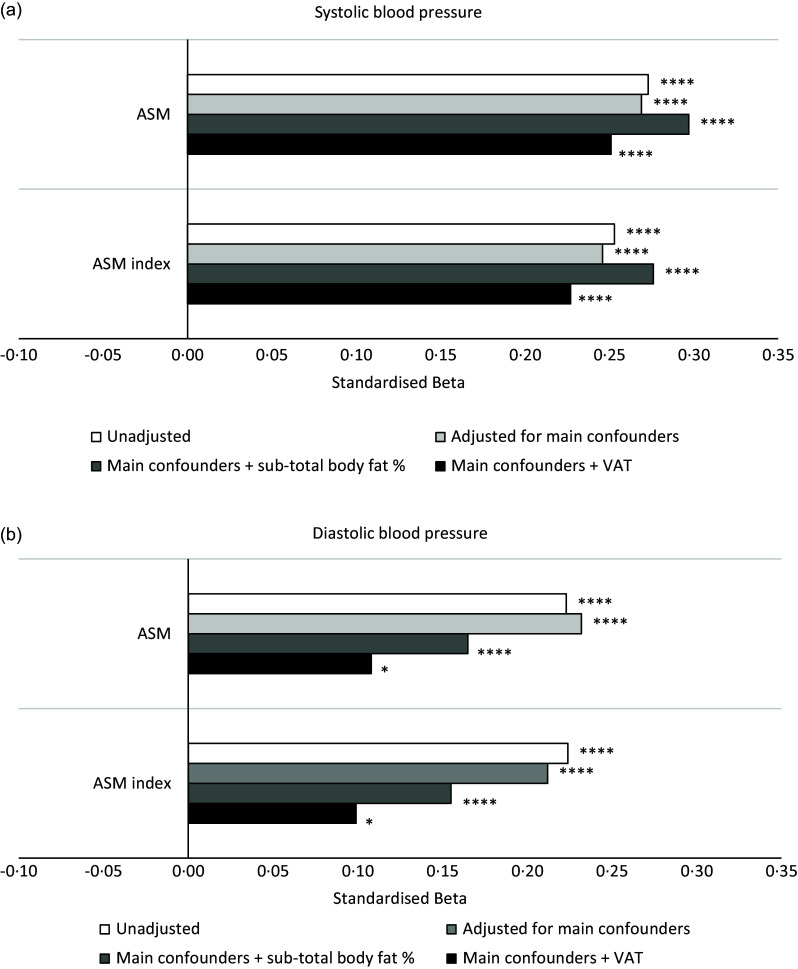
Associations of measures of appendicular skeletal muscle mass (predictors) with systolic (a) and diastolic (b) blood pressure (outcomes). Linear regression models unadjusted; adjusted for main confounders (age, height, smoking, alcohol and HIV status) only; and adjusted for main confounders plus whole-body fat percentage; adjusted for main confounders plus whole-body fat percentage plus VAT (visceral adipose tissue). ****P< 0·0001, *P< 0·05. NB: Height was excluded as a confounder in the models where the ASM (appendicular skeletal muscle mass) index was the predictor because of multicollinearity. Systolic and diastolic blood pressure values were mathematically transformed using a Standardised asinh(x) function in R.

Figure 3 summarises results from linear regression models of associations between measures of ASM (absolute and index) and measures of BP. Both ASM and ASM index were associated with higher systolic and diastolic BP even after adjusting for the main confounders (all *P*< 0·001). The evidence of these associations remained even after adjusting for either whole-body fat percentage (all *P*< 0·001) or VAT (all *P*< 0·015).

### Differences in normalised protein expression between the normal and elevated BP groups

Out of all proteins included in the analysis (*n* 363), only nineteen proteins were different between participants with normal and elevated BP (Table 2). However, after adjusting for multiple testing, there was only sufficient evidence of a difference for REN (FDR-adjusted *P*= 0·008) (Table 2).

**Table 2.** Comparison of normalised protein expressions between the normal and elevated blood pressure groups

Protein	Normalised protein expression		*P*	FDR-adjusted *P*
	Normal BP (*n* 44)	Elevated BP (*n* 44)		
REN	0·400	–0·409	2·03 × 10^−05^	0·008
FABP4	0·968	1·551	0·002	0·376
F9	0·998	1·191	0·004	0·376
ACAN	1·555	1·305	0·004	0·376
ART3	0·871	0·616	0·011	0·672
ROR1	1·487	1·260	0·012	0·672
PAG1	1·428	1·805	0·014	0·672
IL6	–0·025	0·449	0·015	0·672
COMT	3·506	4·004	0·017	0·672
STK11	3·704	4·195	0·019	0·672
PTN	2·997	2·592	0·020	0·672
CTSD	0·503	0·725	0·025	0·779
SLITRK6	1·039	0·836	0·030	0·836
ITGB1BP2	5·019	5·660	0·031	0·836
NPTXR	1·107	0·810	0·042	0·879
COL1A1	1·702	1·474	0·043	0·879
SNX9	3·224	3·596	0·044	0·879
CD163	0·849	1·064	0·044	0·879
GRAP2	6·499	7·139	0·045	0·879

BP, blood pressure; FDR, false discovery rate.

### Associations of the protein biomarkers with measures of BP and body composition

Only protein biomarkers that showed sufficient evidence of association with systolic BP, diastolic BP, VAT and ASM (*P*< 0·050), after including confounders, are presented in Tables 3–5. Table 3 shows that although thirty-seven biomarkers were associated with systolic BP, none of these relationships remained significant after adjusting for multiple testing (FDR-adjusted *P*> 0·050). When exploring associations with diastolic BP, seventeen protein biomarkers remained significant in the models after adjusting for the main confounders. However, only REN was associated with lower diastolic BP after adjusting for multiple testing (FDR-adjusted *P*= 0·005, Table 4).

**Table 3.** Associations between protein biomarkers and systolic blood pressure

	Unadjusted		Adjusted for confounders		
Protein	Beta	*P*	Beta	*P*	FDR-adjusted *P*
CTSL	0·074	0·007	0·088	0·001	0·450
PAG1	0·055	0·005	0·059	0·002	0·317
PTN	–0·052	0·002	–0·046	0·006	0·792
CD46	0·068	0·009	0·069	0·007	0·621
REN	–0·041	0·008	–0·040	0·009	0·672
GPNMB	0·099	0·025	0·109	0·012	0·757
MSMB	0·031	0·050	0·039	0·014	0·742
MTPN	0·060	0·009	0·056	0·015	0·663
TNC	0·033	0·061	0·043	0·017	0·692
LILRB2	0·076	0·037	0·086	0·018	0·666
BLMH	0·057	0·016	0·055	0·020	0·665
TCN2	0·084	0·034	0·093	0·020	0·616
CTSH	0·035	0·045	0·039	0·021	0·596
FCGR2A	0·075	0·036	0·079	0·022	0·583
SORT1	0·067	0·018	0·064	0·023	0·556
GP1BA	0·048	0·048	0·054	0·023	0·526
ADGRE5	0·070	0·028	0·071	0·023	0·502
SCARF1	0·036	0·051	0·041	0·024	0·489
ICAM5	–0·019	0·453	–0·004	0·025	0·479
CD55	0·067	0·056	0·076	0·026	0·479
F9	0·078	0·087	0·099	0·026	0·458
PTPRF	0·069	0·044	0·075	0·028	0·462
PLAT	0·014	0·214	0·026	0·028	0·445
PEAR1	0·055	0·109	0·075	0·029	0·437
NPTXR	–0·056	0·007	–0·045	0·030	0·439
VWF	0·024	0·032	0·024	0·030	0·425
HSPG2	0·045	0·114	0·060	0·032	0·436
ITGB2	0·049	0·031	0·049	0·033	0·431
ITGB1	0·052	0·059	0·059	0·033	0·419
FABP4	0·030	0·055	0·034	0·033	0·406
CHI3L1	0·028	0·083	0·034	0·034	0·401
CORO1A	0·023	0·058	0·024	0·038	0·432
LILRA5	0·061	0·044	0·060	0·043	0·478
NPDC1	–0·031	0·019	–0·027	0·045	0·479
CD69	0·022	0·055	0·023	0·047	0·486
IL6ST	0·092	0·119	0·114	0·047	0·476
STK11	0·032	0·028	0·029	0·047	0·468

Linear regression models showing associations between protein biomarkers (predictors) and systolic blood pressure (outcome). Only models with sufficient evidence of association (*P*< 0·050), after including the potential confounders, are shown. Potential confounders included age, height, smoking, alcohol and HIV status). Systolic blood pressure values were mathematically transformed using a Standardised asinh(x) function in R. Beta, beta coefficient; FDR, false discovery rate.

**Table 4.** Associations between protein biomarkers and diastolic blood pressure

	Unadjusted		Adjusted for confounders		
Protein	Beta	*P*	Beta	*P*	FDR-adjusted *P*
REN	–0·062	2·24 × 10^−05^	–0·061	1·47 × 10^−05^	0·005
PAG1	0·056	0·004	0·060	0·001	0·173
CTSD	0·077	0·010	0·074	0·013	1·000
NT-proBNP	0·017	0·045	0·020	0·017	1·000
ITGB1BP2	0·029	0·004	0·024	0·017	1·000
F9	0·098	0·028	0·098	0·021	1·000
USP8	0·026	0·004	0·021	0·023	1·000
SLITRK6	–0·075	0·020	–0·068	0·026	1·000
FABP4	0·037	0·017	0·033	0·027	1·000
MPHOSPH8	0·029	0·009	0·025	0·030	1·000
NADK	0·037	0·014	0·032	0·031	1·000
SNX9	0·039	0·016	0·034	0·033	1·000
CDH5	–0·055	0·058	–0·059	0·034	0·962
PLAT	0·016	0·154	0·024	0·036	0·939
COMT	0·037	0·009	0·029	0·041	0·989
VAMP5	0·036	0·052	0·040	0·043	0·981
FCGR3B	0·032	0·171	0·045	0·043	0·924

Linear regression models showing associations between protein biomarkers (predictors) and diastolic blood pressure (outcome). Only models with sufficient evidence of association (*P*< 0·050), after including the potential confounders, are shown. Potential confounders included age, height, smoking, alcohol and HIV status). Diastolic blood pressure values were mathematically transformed using a Standardised asinh(x) function in R. Beta, beta coefficient; FDR, false discovery rate.

**Table 5.** Associations between protein biomarkers and visceral adipose tissue

	Unadjusted		Adjusted for confounders		
Protein	Beta	*P*	Beta	*P*	FDR-adjusted *P*
LEP	0·607	< 2 × 10^−16^	0·608	< 2 × 10^−16^	7·32 × 10^−14^
FABP4	0·776	3·31 × 10^−14^	0·790	3·73 × 10^−14^	6·83 × 10^−12^
IL6	0·549	5·10 × 10^−07^	0·523	2·85 × 10^−06^	3·48 × 10^−04^
NOTCH3	–0·756	1·82 × 10^−05^	–0·749	2·20 × 10^−05^	0·002
ACAN	–1·190	1·09 × 10^−06^	–1·170	2·68 × 10^−05^	0·002
NCAM1	–0·900	1·71 × 10^−05^	–0·879	4·15 × 10^−05^	0·003
ART3	–0·838	1·36 × 10^−04^	–0·885	1·13 × 10^−04^	0·006
PLA2G1B	–0·658	1·40 × 10^−04^	–0·639	2·52 × 10^−04^	0·012
COL1A1	–0·786	5·71 × 10^−05^	–0·742	0·001	0·025
DKK3	–0·767	2·50 × 10^−04^	–0·787	0·001	0·023
CELA3A	–0·428	0·001	–0·452	0·001	0·022
ENG	–1·357	0·001	–1·349	0·001	0·020
NPTXR	–0·534	4·50 × 10^−04^	–0·514	0·001	0·026
GGH	0·784	0·001	0·754	0·001	0·033
AMY2B	–0·511	0·006	–0·607	0·002	0·044
CNTN1	–0·806	0·001	–0·755	0·002	0·043
AMY2A	–0·551	0·007	–0·651	0·002	0·050
MSTN	–0·454	0·003	–0·456	0·004	0·081
DDC	–0·375	0·005	–0·389	0·004	0·082
IGFBP1	–0·177	0·003	–0·174	0·004	0·081
CST6	–0·480	0·007	–0·528	0·005	0·081
PDGFRA	–0·726	0·002	–0·668	0·005	0·089
LDLR	0·391	0·013	0·451	0·005	0·085
ADGRG2	–0·813	0·005	–0·794	0·006	0·098
APLP1	–0·340	0·002	–0·312	0·006	0·095
TGFBR3	–0·255	0·020	–0·308	0·007	0·101
PLTP	–0·478	0·013	–0·524	0·007	0·102
ANGPTL1	–0·622	0·012	–0·660	0·008	0·104
KITLG	–0·448	0·009	–0·456	0·008	0·101
LGALS3	0·658	0·006	0·653	0·009	0·114
LTBP2	–0·433	0·043	–0·575	0·010	0·115
BOC	–0·418	0·018	–0·459	0·010	0·114
CNDP1	0·261	0·035	0·328	0·011	0·118
TFPI	–0·483	0·003	–0·423	0·012	0·126
LEPR	–0·605	0·017	–0·644	0·012	0·128
ENTPD6	–0·360	0·038	–0·440	0·015	0·147
PRSS27	–0·388	0·021	–0·424	0·015	0·149
NOTCH1	–0·978	0·016	–0·983	0·016	0·153
THBD	–0·720	0·011	–0·699	0·016	0·154
CDHR5	0·386	0·021	0·408	0·017	0·158
PTPRS	–0·750	0·009	–0·692	0·018	0·158
F9	0·812	0·015	0·813	0·018	0·154
SFTPD	–0·217	0·063	–0·279	0·019	0·162
ROR1	–0·598	0·016	–0·590	0·020	0·166
PTGDS	–0·662	0·012	–0·626	0·020	0·163
IGFBP2	–0·290	0·015	–0·273	0·023	0·179
FUCA1	–0·375	0·036	–0·422	0·023	0·177
VCAM1	–0·903	0·009	–0·800	0·023	0·175
LILRA5	0·527	0·020	0·513	0·023	0·172
PEAR1	–0·696	0·006	–0·591	0·025	0·181
AOC3	–0·555	0·016	–0·520	0·026	0·186
PTN	–0·301	0·020	–0·291	0·027	0·188
CHI3L1	0·272	0·022	0·271	0·028	0·195
FBP1	0·124	0·017	0·115	0·029	0·197
CBLIF	–0·324	0·027	–0·330	0·031	0·203
THPO	–0·233	0·062	–0·275	0·031	0·200
COL4A1	–0·343	0·008	–0·290	0·031	0·198
FCN2	0·431	0·023	0·424	0·031	0·197
ANG	–0·673	0·018	–0·623	0·032	0·195
CTSB	–0·303	0·028	–0·300	0·033	0·199
CTSD	0·503	0·028	0·515	0·033	0·197
APOM	–0·475	0·028	–0·460	0·037	0·216
HMOX1	–0·169	0·106	–0·237	0·038	0·222
HYAL1	–0·527	0·048	–0·556	0·039	0·225
IGFBPL1	–0·475	0·041	–0·488	0·040	0·222
GP2	–0·203	0·090	–0·248	0·040	0·223
NID1	–0·511	0·023	–0·464	0·041	0·225
PAM	–0·602	0·038	–0·608	0·041	0·222
CLEC5A	–0·391	0·048	–0·413	0·042	0·220
IGSF8	–0·378	0·054	–0·411	0·042	0·220
FABP6	–0·272	0·036	–0·266	0·042	0·219
DCN	–0·508	0·077	–0·590	0·042	0·216
GHRL	–0·125	0·071	–0·146	0·043	0·217
TNF	–0·170	0·020	–0·147	0·048	0·237
ADH4	0·196	0·033	0·200	0·049	0·241
GSTA1	0·206	0·061	0·229	0·049	0·237

Linear regression models showing associations between protein biomarkers (predictors) and visceral adipose tissue (outcome). Only models with sufficient evidence of association (*P*< 0·050), after including the confounders, are shown. Confounders included age, height, smoking, alcohol and HIV status). Visceral adipose tissue values were mathematically transformed using a box-cox function in R. Beta, beta coefficient.

Tables 5 and 6 summarise linear regression models for the associations of the protein biomarkers with VAT (*n* 76) and ASM (*n* 44), respectively, before and after adjusting for the main confounders and multiple testing. After adjusting for multiple testing, four proteins (LEP, FABP4, IL6 and GGH) were positively associated with both VAT and ASM (all FDR-adjusted *P*< 0·050). Likewise, four other proteins (ACAN, CELA3A, PLA2G1B and NCAM1) were inversely associated with these two outcomes. Table 5 also shows that eight other proteins (NOTCH3, ART3, COL1A1, DKK3, ENG, NPTXR, AMY2B and CNTN1) were associated with lower VAT only (all FDR-adjusted *P*< 0·050). Table 6 shows that only IGFBP1 was associated with lower ASM after adjusting for multiple testing (FDR-adjusted *P*= 0·009).

**Table 6.** Associations between protein biomarkers and appendicular skeletal muscle mass

	Unadjusted		Adjusted for confounders		
Protein	Beta	*P*	Beta	*P*	FDR-adjusted *P*
LEP	0·483	6·43 × 10^−10^	0·452	1·50 × 10^−09^	5·49 × 10^−07^
FABP4	0·618	1·64 × 10^−08^	0·609	6·22 × 10^−09^	1·14 × 10^−06^
ACAN	–0·971	1·05 × 10^−04^	–1·179	5·45 × 10^−06^	0·001
CELA3A	–0·493	1·29 × 10^−04^	–0·506	3·62 × 10^−05^	0·003
PLA2G1B	–0·602	0·001	–0·649	6·79 × 10^−05^	0·005
NCAM1	–0·702	0·001	–0·794	8·85 × 10^−05^	0·005
IL6	0·391	0·001	0·419	9·26 × 10^−05^	0·005
IGFBP1	–0·233	6·05 × 10^−05^	–0·209	2·06 × 10^−04^	0·009
GGH	0·732	0·002	0·791	2·82 × 10^−04^	0·011
COMP	0·930	1·48 × 10^−04^	0·761	0·001	0·050
ART3	–0·475	0·036	–0·653	0·003	0·098
AMY2A	–0·302	0·145	–0·596	0·003	0·091
GP2	–0·356	0·002	–0·331	0·003	0·086
AMY2B	–0·258	0·172	–0·536	0·004	0·092
APOM	–0·466	0·031	–0·590	0·004	0·095
DKK3	–0·338	0·118	–0·606	0·006	0·129
LEPR	–0·726	0·004	–0·660	0·006	0·131
PEAR1	–0·594	0·021	–0·653	0·008	0·161
LDLR	0·409	0·010	0·401	0·009	0·165
ADGRG2	–0·634	0·029	–0·716	0·009	0·165
CBLIF	–0·229	0·120	–0·370	0·009	0·164
SERPINB5	–0·187	0·059	–0·245	0·009	0·158
COL1A1	–0·494	0·014	–0·531	0·010	0·163
TFPI	–0·418	0·010	–0·399	0·011	0·173
IGFBP2	–0·282	0·018	–0·283	0·011	0·167
PRSS2	–0·293	0·050	–0·384	0·012	0·164
REG1B	–0·381	0·013	–0·408	0·013	0·176
ADAMTS13	–0·306	0·137	–0·504	0·016	0·205
SSC4D	0·138	0·034	0·151	0·016	0·204
CCL5	–0·214	0·017	–0·200	0·020	0·249
ENG	–0·684	0·088	–0·853	0·025	0·291
COL4A1	–0·236	0·071	–0·283	0·025	0·286
LGALS3	0·556	0·022	0·531	0·025	0·281
NOTCH3	–0·348	0·059	–0·379	0·028	0·301
DDC	–0·319	0·019	–0·280	0·030	0·314
REG1A	–0·379	0·024	–0·360	0·038	0·390
ANG	–0·482	0·094	–0·563	0·039	0·385
ESAM	–0·275	0·132	–0·356	0·039	0·380
NPTXR	–0·308	0·048	–0·305	0·040	0·379
THBS4	0·387	0·056	0·394	0·040	0·370
NID1	–0·380	0·093	–0·432	0·043	0·385
CPB1	–0·203	0·095	0·241	0·044	0·385
MB	0·452	0·010	0·347	0·048	0·407
HYAL1	–0·335	0·211	–0·500	0·049	0·404

Linear regression models showing associations between protein biomarkers (predictors) and appendicular skeletal muscle mass (outcome). Only models with sufficient evidence of association (*P*< 0·050), after including the potential confounders, are shown. Potential confounders included age, height, smoking, alcohol and HIV status). Total appendicular skeletal muscle mass (ASM) values were mathematically transformed using a box-cox function in R. Beta, beta coefficient; FDR, false discovery rate.

## Discussion

Recent findings in populations of non-African ancestry have demonstrated that skeletal muscle mass was associated with higher BP, suggesting that having higher muscle mass may not always yield positive health outcomes. In the present study, we confirmed that ASM was associated with higher systolic and diastolic BP in young adult black South African women and that these associations were independent of overall and central adiposity. Further, using a targeted proteomics approach, we also demonstrated that renin (REN) expression was lower in women with elevated BP and associated with lower diastolic BP, but not systolic BP, and we identified several proteins that were associated with both ASM and VAT. The study did not find sufficient evidence to suggest that the associations between measures of body composition and BP were mediated by any of the protein biomarkers measured.

The relationship between skeletal muscle mass and BP has been investigated in many studies with contradictory results^(9,13,18,20–22)^. Many of the previous studies were conducted in predominantly older participants who were already at increased CVD risk, and often the studies failed to test whether these relationships were independent of body fat^(9,13,18,20–22)^. Our observation that ASM was associated with higher systolic and diastolic BP is in accordance with most of these previous findings^(13,18,21,22)^. For example, the multi-ancestry study that included both young and middle-aged adults (median age of 36 years) also suggested that skeletal muscle mass was positively associated with BP independent of body fat mass^(22)^. The few studies that suggested an inverse relationship between measures of skeletal muscle mass and BP either failed to adjust for height, which is key in accounting for body size, or only focused on hypertension rather than the specific BP measurements^(9,20)^.

The relationship between skeletal muscle mass and higher BP is well acknowledged and may be influenced by many physiological factors including metabolic activity. For example, higher skeletal muscle mass leads to higher BP due to greater demand on the heart to pump blood through a larger body mass, which increases the workload on the heart and arteries^(37)^. Additionally, the muscle tissue is metabolically active and can produce substances like cytokines and other metabolites, which can have a direct effect on blood vessels^(38)^. These substances can cause vasoconstriction, which increases the resistance to blood flow and ultimately increases BP and CVD risk^(39)^. Accordingly, a recent study that included young adult men and women of European ancestry showed that skeletal muscle gain was associated with markers of increased CVD risk, including increased atherogenic substances such as VLDL-cholesterol^(40)^.

The above potential mechanisms are also in line with our observations that the associations between ASM and BP were independent of measures of body fat and its distribution. The notion that skeletal muscle mass is associated with higher BP independent of body fat had been suggested by some studies conducted in European children^(24,25)^. To the best of our knowledge, we have shown this for the first time in women of African ancestry, who are known to have a unique body composition phenotype and a higher risk of developing CVD^(27)^. Notably, a recent multi-ethnic study, comprising a small portion of young and middle-aged Africans, also demonstrated that skeletal muscle mass was associated with higher BP independent of body fat^(22)^. Findings from that study further suggested that trunk (central) fat mass was the major contributor (38–61 %) to both higher systolic and diastolic BP, while ASM was a relatively major contributor (35–43 %) to higher systolic BP only^(22)^. Our observations were partially in line with these previous findings, as we demonstrated that the associations between overall and central body fat with diastolic BP were independent of ASM, while the associations with systolic BP were not. This suggests a different mechanism for systolic BP and warrants further investigations to understand the confounding role of skeletal muscle mass.

Due to the multifactorial nature of the regulation of both skeletal muscle mass and BP, the mechanisms involved in the influence of skeletal muscle mass on systolic and diastolic BP are complex^(41)^. To identify proteins that may mediate the association between body composition and BP, we investigated 363 protein biomarkers that are related to cardiometabolic disease risk. The biomarkers included those involved in the inflammatory response and regulation of body weight, some of which are known to influence skeletal muscle mass and BP^(42,43)^. In our study, we only identified one protein biomarker, REN, that was associated with BP, and as this was not associated with skeletal muscle mass, there was not sufficient evidence to support our hypothesis that the associations between skeletal muscle mass and BP are mediated by the included proteins.

The association of REN with lower diastolic BP but not with body composition suggested that the influence of renin on BP is independent of body composition. REN plays a crucial role in the renin-angiotensin-aldosterone system (RAAS), a complex system that regulates BP^(44)^. Traditionally, plasma REN concentrations were expected to associate with higher BP in all populations. This is because the role of REN is to initiate a cascade of events that ultimately lead to increased BP, specifically, by increasing the production of angiotensin II – which narrows blood vessels, and the production of aldosterone – which promotes sodium retention^(44)^. However, studies conducted in populations of African ancestry have consistently reported an inverse association between circulating REN and BP^(45–47)^. The ethnic disparity in the relationship between REN levels and BP is thought to be complex and likely to involve interactions between genetic, environmental and lifestyle factors^(48)^. For example, some studies in populations of African ancestry have reported a higher frequency of genetic variants associated with increased salt sensitivity^(49)^. Accordingly, increased salt sensitivity is common in Africans^(50)^.

Since our proteins were measured in circulation, urinary or tissue-specific RAAS components might show different results. Previous studies have shown that tissue-specific RAAS activity can vary significantly from circulating levels, which may provide additional insights into localised BP regulation^(44)^. While circulating REN can offer some approximation of renal/urinary RAAS activation, it is not a direct measure. Renal-specific measurements would be necessary to fully understand the local RAAS activity and its impact on BP^(45)^. Future studies should consider both systemic and local RAAS components in understanding BP regulation in the context of body composition, particularly in different populations.

All the protein biomarkers that were associated with higher central body fat (VAT) were also associated with higher skeletal muscle mass. The positive associations of circulating LEP (leptin), FABP4 (fatty acid binding protein 4) and IL6 with both VAT and ASM were expected as the physiological pathways in the regulation of these two components of body composition are established. LEP is a hormone that is primarily produced by fat cells and signals the brain about energy storage levels^(51)^, while this hormone is also known to influence skeletal muscle growth^(52)^. FABP4, also strongly associated with VAT and ASM, has a role in promoting lipogenesis in skeletal muscle cells by activating the PPAR *γ* signalling pathway^(53)^, while IL6, a well-acknowledged cytokine that participates in inflammation and B cell maturation^(54)^, has a role in promoting hypertrophic skeletal muscle growth^(55)^. While there are potential physiological pathways by which the enzyme gamma-glutamyl hydrolase (GGH) could influence VAT and skeletal muscle mass, the specific relationships are not established. GGH plays a role in folate metabolism and thus indirectly influences the synthesis of both amino acids and nucleotides – which are important in cellular growth and replication^(56)^. However, further research is still required to elucidate the role of GGH in fat and muscle cell growth.

Similarly, limited physiological functions of the protein biomarkers (aggrecan: ACAN; chymotrypsin-like elastase 3A: CELA3A; phospholipase A2 group IB: PLA2G1B; and neural cell adhesion molecule 1: NCAM1) that were associated with lower VAT and ASM in the present study have been reported. However, the direct roles of these proteins in these two outcomes are unclear. While ACAN contributes to cartilage structure and joint function^(57)^, NCAM1 plays a role in multiple neuronal functions, including neurite outgrowth, synapse formation, maturation and plasticity^(58)^. Conversely, both CELA3A and PLA2G1B are involved in digestion. While CELA3A cleaves proteins after alanine residues^(59)^, PLA2G1B hydrolyses phospholipids to promote lipid digestion and absorption^(60)^. Further investigations are still required to understand why these proteins associate with both lower VAT and skeletal muscle mass.

The study found several proteins associated with lower VAT only. These proteins have diverse physiological functions^(61–65)^ and included pancreatic *α*-amylase (AMY2B), which aids in digestion and may indirectly affect adiposity^(61)^. Neurogenic locus notch homolog protein 3 (NOTCH3) inhibits adipogenesis^(66)^, while ADP-ribosyltransferase 3 (ART3) is involved in adipocyte differentiation and lipid storage^(62)^. Collagen1a1 (COL1A1) is an extracellular matrix protein with lower levels in obesity, suggesting a role in adipose tissue development^(67)^. Dickkopf-3 (DKK3) and endoglin (ENG) may influence adipose tissue by regulating tissue development pathways^(63,64)^. Neuronal pentraxin receptor (NPTXR) and contactin 1 (CNTN1) may affect adiposity through neural circuits controlling energy balance and feeding behaviour^(65)^.

The only protein that was associated with lower skeletal muscle mass was IGFBP1 (insulin-like growth factor binding protein 1). This relationship is well known and was reported in our recent study of middle-aged black South African men and women^(31)^. IGFBP1 binds to IGF (insulin-like growth factors) with high affinity and reduces the availability of free IGF that can interact with the IGF1 receptor, hindering muscle growth and maintenance^(68)^.

### Study strengths and limitations

This is a cross-sectional study from which causality cannot be inferred. The generalisation of our findings should be limited to populations of African ancestry, as ethnic differences in body composition and cardiometabolic disease risk are well known^(27)^. The use of a targeted proteomics approach could have potentially excluded biomarkers that may be relevant to the complex relationships between body composition and BP. Our power analysis only considered the effect size in the relationship between skeletal muscle mass and systolic BP. Another key limitation of this study was the absence of data on physical activity. Physical activity is a crucial factor that significantly influences both body composition and BP. Without these data, our ability to fully understand the interplay between these variables is limited. Future studies should aim to include detailed physical activity measurements to provide a more comprehensive analysis of these relationships. Regardless of these limitations, this is the first study to investigate whether the association between measures of muscle and fat and BP is independent of the other component of body composition in a population of African ancestry. The study’s use of a target proteomics approach that included 363 proteins has provided some insights into the potential pathways involved in body composition and BP in this population.

### Conclusions

We report that measures of overall and central adiposity and skeletal muscle mass were associated with higher systolic and diastolic BP in young adult black South African women. This suggests a need for future interventions that aim to increase muscle mass to also monitor BP in this population. We also demonstrated that the associations between measures of muscle mass and BP were independent of whole-body fat and VAT, suggesting a distinct role of muscle mass in increasing BP. We have also shown that renin expression was associated with lower diastolic BP, but not systolic BP in this population. Although we have also identified several proteins that were associated with skeletal muscle mass and VAT, none of these proteins were associated with BP.
